# Elevated Hapln2 Expression Contributes to Protein Aggregation and Neurodegeneration in an Animal Model of Parkinson's Disease

**DOI:** 10.3389/fnagi.2016.00197

**Published:** 2016-08-23

**Authors:** Qinqin Wang, Qinbo Zhou, Shuzhen Zhang, Wei Shao, Yanqing Yin, Yandong Li, Jincan Hou, Xinhua Zhang, Yongshun Guo, Xiaomin Wang, Xiaosong Gu, Jiawei Zhou

**Affiliations:** ^1^Institute of Neuroscience, State Key Laboratory of Neuroscience, Chinese Academy of Sciences Center for Excellence in Brain Science and Intelligence Technology, Shanghai Institutes for Biological Sciences, Chinese Academy of SciencesShanghai, China; ^2^University of Chinese Academy of SciencesShanghai, China; ^3^Co-innovation Center of Neuroregeneration, School of Medicine, Nantong UniversityNantong, China; ^4^Center of Parkinson's Disease, Beijing Institute for Brain DisordersBeijing, China

**Keywords:** hyaluronan and proteoglycan binding link protein 2, Parkinson's disease, dopaminergic neurons, ubiquitin-proteasome system, aggregation

## Abstract

Parkinson's disease (PD), the second most common age-associated progressive neurodegenerative disorder, is characterized by the loss of dopaminergic (DA) neurons in the substantia nigra pars compacta (SN). The pathogenesis of PD and the mechanisms underlying the degeneration of DA neurons are still not fully understood. Our previous quantitative proteomics study revealed that hyaluronan and proteoglycan binding link protein 2 (Hapln2) is one of differentially expressed proteins in the substantia nigra tissues from PD patients and healthy control subjects. However, the potential role of Hapln2 in PD pathogenesis remains elusive. In the present study, we characterized the expression pattern of Hapln2. *In situ* hybridization revealed that Hapln2 mRNA was widely expressed in adult rat brain with high abundance in the substantia nigra. Immunoblotting showed that expression levels of Hapln2 were markedly upregulated in the substantia nigra of either human subjects with Parkinson's disease compared with healthy control. Likewise, there were profound increases in Hapln2 expression in neurotoxin 6-hydroxydopamine-treated rat. Overexpression of Hapln2 *in vitro* increased vulnerability of MES23.5 cells, a dopaminergic cell line, to 6-hydroxydopamine. Moreover, Hapln2 overexpression led to the formation of cytoplasmic aggregates which were co-localized with ubiquitin and E3 ligases including Parkin, Gp78, and Hrd1 *in vitro*. Endogenous α-synuclein was also localized in Hapln2-containing aggregates and ablation of Hapln2 led to a marked decrease of α-synuclein in insoluble fraction compared with control. Thus, Hapln2 is identified as a novel factor contributing to neurodegeneration in PD. Our data provides new insights into the cellular mechanism underlying the pathogenesis in PD.

## Introduction

Parkinson's disease (PD) is the second most common neurodegenerative disorder affecting more than 1% of the people aged over 65 years around the world (Delenclos et al., 2016). It is mainly characterized by severe movement symptoms including resting tremor, slowness of movement, rigidity and postural instability (Kadoguchi et al., 2014; Delenclos et al., 2016). Recent studies have shown that a number of significant non-motor symptoms, such as depression, anxiety, sleep disturbance, and pain, are also evident which appear to have adverse impact on quality of life of patient with PD (Martinoia et al., 2005). The economic burden of PD is projected to grow substantially over the next few decades as the size of the elderly population grows (Hirakawa et al., 2000). Pathologically, PD is marked by the progressive loss of dopaminergic (DA) neurons in the ventral midbrain and the formation of α-synuclein-containing aggregates in neuronal cell bodies and neuritis (Anderson et al., 2006; Kadoguchi et al., 2014). Although a large variety of possible pathogenic factors, including excessive release of oxygen free radicals during enzymatic dopamine breakdown, impairment of mitochondrial function and calcium homeostasis, dysregulation of LRRK2 kinase, neuroinflammation and loss of trophic support, have been reported to contribute to the disease processes, the etiology of PD has not yet been fully understood (Block and Hong, 2005; Dick et al., 2007; Paumier et al., 2015; Von Stockum et al., 2015), indicating the urgent need to identify target of PD pathogenesis.

Emerging evidence indicates that abnormalities of some PD related proteins, such as α-synuclein and parkin, play vital roles during the progression of PD (Mukherjee et al., 2015; Oliveira et al., 2015). Neural progenitor cell lines from PD patient with α-synuclein overexpression exhibited a reduced ability to differentiate into DA neurons (Oliveira et al., 2015). Moreover, overexpression of wild-type or A53T mutated α-synuclein by recombinant adeno-associated virus mediated-overexpression in the rat substantia nigra (SN) induced the loss of DA neurons and the formation of α-synuclein aggregation (Lu et al., 2015). Interestingly, Parkin deficiency facilitated the cell-to-cell transmission of α-synuclein (Cha et al., 2015). These results suggest that investigation of the proteins involved in the aggregates formation and in the neurodegeneration of nigral DA neurons will provide new insights into the pathogenesis of PD and may help identify new therapeutic targets.

In our previous efforts to characterize the proteome profiles of nigral tissue of PD patients, we found that there were 11 proteins that showed significant alteration in expression levels in the SN between the two cohorts (Liu et al., 2015). Among the 11 proteins, Hapln2 (also known as brain-derived link protein 1, Bral1) showed dramatic upregulation in the brain tissues of PD patients compared with the healthy controls. Hapln2 is one of the hyaluronan and proteoglycan binding link proteins, which is consisted of four members: Hapln1 (also known as cartilage link protein, Crtl1), Hapln2 (Bral1), Hapln3, and Hapln4 (Bral2) (Spicer et al., 2003). Among these four link proteins, Hapln2 and Hapln4 are preferentially expressed in the central nervous system (Spicer et al., 2003). Hapln2 is important for stabilizing and enhancing the binding of the four CSPG core proteins including versican, BCAN, aggrecan, and NCAN to hyaluronan (Spicer et al., 2003; Kähler et al., 2011).

The human HAPLN2 is encoded by four exons, consisting of one immunoglobulin (Ig) fold and two proteoglycan tandem repeat (PTR) domains (Hirakawa et al., 2000). Northern blot analysis showed that Hapln2 mRNA was detected solely in the adult human and mouse brains (Hirakawa et al., 2000). Immunostaining also revealed that the expression of Hapln2 started at P20 in mice when axonal myelination took place in the white matter and was predominantly expressed in myelinated fiber tracts in the adult brain (Oohashi et al., 2002). As one of the hyaluronan and proteoglycan binding link proteins predominantly expressed in myelinated fiber tracts in the adult brain, Hapln2 has been reported to play an important role in maintaining and enhancing extracellular matrix (ECM) structures and function in the formation of diffusion barrier around the nodes of Ranvier and conduction velocity in mouse brain (Spicer et al., 2003; Bekku et al., 2010; Kähler et al., 2011). Hapln2 was co-localized with versican at the nodes of Ranvier in the myelinated white matter (Oohashi et al., 2002). Previous results showed that Hapln2-deficient mice exhibited abnormal expression pattern of hyaluronan-associated ECM and decrease of nerve conduction speed in the brain, indicating the vital role of Hapln2 for neuronal conductivity (Bekku et al., 2009, 2010). Moreover, accumulating evidence indicates that Hapln2 is associated with brain disorders. The Hapln2 expression levels were remarkably reduced in schizophrenia anterior temporal lobe (Neumann et al., 2009), while Hapln2 has been recently shown to be accumulated in the neurofibrillary tangle of Alzheimer's brain (Bandopadhyay, 2016). However, the role of Hapln2 in the degeneration of DA neurons during PD pathogenesis is totally unknown.

In the present study, we verified the upregulation of Hapln2 in human PD brains using western blot. We also found that Hapln2 was markedly upregulated in the SN of 6-OHDA-induced rat PD model. Overexpression of Hapln2 resulted in a profound cell death of primary cultured neurons and MES23.5 cells *in vitro*. Besides, immunofluorescence revealed that overexpression of Hapln2 induced aggregates formation in which α-synuclein is accumulated. Moreover, Hapln2 ablation led to a significant decrease of the ratio of insoluble form to insoluble form of α-synuclein in mouse brain. Our data demonstrated that Hapln2 is involved in PD pathogenesis and the role of Hapln2 in PD may be associated with ubiquitin-proteasome pathway (UPP).

## Materials and methods

### Human tissue collection

Fresh-frozen ventral mesencephalic tissues were obtained from the Netherlands Brain Bank, Netherlands Institute for Neuroscience, Amsterdam, the Netherlands. All the materials have been collected from donors for or from whom a written informed consent for a brain autopsy and the use of the material and clinical information for research purposes had been obtained by the Netherlands Brain Bank. Frozen brain tissues from three pairs of age and gender-matched PD patients and healthy controls were used. Summary of the demographic and clinicopathological data on the three PD and three control cases used in this study were previously described by Liu et al. (2015).

### Animals

Adult Sprague–Dawley rats (250–280 g) were purchased from the Shanghai Laboratory Animal Center, Chinese Academy of Sciences. Hapln2 knockout mice were a kind gift from Dr. T. Oohashi (Bekku et al., 2010). The animals were maintained on a 12 h light/dark cycle at 23°C with food and water available *ad libitum*. All the procedures were approved by the Institutional Animal Care and Use Committee and were in accordance with the US National Institutes of Health Guide for the Care and Use of Laboratory Animals.

### The 6-OHDA-induced rat PD model

The 6-OHDA-induced rat model of PD was established as described previously (Zhou et al., 2011). In brief, the adult rats under deep anesthesia received a bilateral injection of 4 μl of 6-OHDA (5 μg/μl in sterile saline) in the striatum using the coordinates relative to bregma (AP: +1.0 mm, ML: −3.0 mm, DV: −5.0 mm) with a Hamilton 10 μl syringe at a rate of 0.5 μl/min. The sham-lesioned rats served as controls were injected with 4 μl of sterile saline in the same region. The needle was withdrawn slowly 5 min following the completion of injection. SN tissues were isolated for western blotting or quantitative PCR 2 or 4 weeks post-surgery.

### *In situ* hybridization

*In situ* hybridization (ISH) was performed on cryosections (15 μm thick) with digoxigenin-labeled single-stranded RNA probes as described previously (Zhou et al., 2011). Briefly, the brain sections were fixed overnight in 4% paraformaldehyde at 4°C. To prepare the Hapln2 (GenBank accession number AB049056) hybridization probes, primers were designed to amplify a fragment with 300–400 bp. Sequences of the primers used were as follow: forward, 5′-ACTAAGGCGTGCCTA TCAACT; reverse, 5′-CCA CTTTGCTCCAGCGTAC -3′. In some cases, brain sections were further processed for tyrosine hydroxylase (TH, a marker for DA neurons) immunohistochemistry using 3.3-diaminobenzidine (DAB, Sigma-Aldrich, St. Louis, MO, USA) as substrate following Hapln2 ISH. The sense probe was used as a negative control.

### RNA isolation and quantitative RT-PCR

Isolation of total RNA was performed as described previously (Shao et al., 2013). Briefly, total RNA was isolated from brain tissues using TRIzol reagent (Invitrogen). Template cDNA was synthesized from 1 μg of extracted total RNA using PrimeScript kit (TaKaRa, Japan) according to the manufacturer's instruction. Quantitative RT-PCR was carried out with SYBR-Green premix Ex *Taq* (Takara, Japan) and detected by a Real Time PCR System (Roche Light Cycler 480 or Rotorgene 6000, USA). Fold changes were calculated using relative quantification methods with β-actin as an internal control gene. The primers were designed using Primer Picking Program and their sequences were as follows: Hapln2, forward, 5′-ACTAAGGCG TGCCTATCAACT-3′, reverse, 5′-CCACTT TGCTCCAGCGTAC-3′; β-actin, forward, 5′-ACCCGCCACCAG TTCGCCAT-3′, reverse, 5′-CTAGGGCGG CCCACGATGGA-3′.

### Extraction of RIPA-soluble and RIPA-insoluble protein fractions of mouse brain

Extraction of RIPA-soluble and RIPA-insoluble protein fractions from brain tissues was described previously (Gallardo et al., 2008; Neumann et al., 2009; Walker et al., 2015; Bandopadhyay, 2016). Previous studies indicated that separation of insoluble fraction was performed on older mice at least 6 months of age (Chandra et al., 2005; Ho et al., 2008). Six-month-old Hapln2 knockout or control mice were injected with LPS (5 mg/kg, i.p. Sigma-Aldrich) in order to enhance protein aggregation. The brain tissues were isolated 24 h after LPS treatment and stored at −20°C prior to use. Frozen brain tissues were thawed on ice and homogenized in 2 × v/w RIPA buffer (150 mM NaCl, 1% Triton X-100, 0.1% SDS, 0.5% sodium deoxycholate, 50 mM NaF, 1 mM EDTA,50 mM Tris, pH 7.5). The lysates was centrifuged at 14,000 g at 4°C for 30 min and the supernatants were collected as the RIPA-soluble fraction. The pellet was washed with RIPA buffer and centrifuged at 14,000 g at 4°C for 10 min for 3 times. The pellet was dissolved in 0.5 × v/w urea buffer (8 M urea, 100 mM NaCl, 1 Mm EDTA, 50 mM Tris, PH 8) as urea-soluble fraction.

### Western blot and quantification

Western blotting was performed following standard procedures as described previously (Li et al., 2006). The primary antibodies used were detailed as follows: mouse monoclonal antibody against Hapln2 (1:2000; Abnova); mouse monoclonal antibody against β-actin (1:5000; Sigma-Aldrich); mouse anti-α-tublin antibody (1:5000; Sigma-Aldrich). The membrane was washed and incubated for 1 h at room temperature with the corresponding secondary antibodies: horseradish peroxidase (HRP)-conjugated goat anti-mouse IgG (1:10,000; Jackson ImmunoResearch Laboratories, USA). Peroxidase activity was detected with SuperSignal WestPico chemiluminescent substrate (Pierce Biotechnology, USA). Immunoreactive bands were visualized and digitized with ImageQuant (LAS-4000, Fujifilm, Japan). Optical densities of bands were analyzed using ImageJ software (NIH). The protein levels were normalized to loading control of β-actin or α-tublin.

### Immunofluorescence and immunohistochemistry

Immunofluorescence was performed as described previously (Shao et al., 2013). Briefly, cryosections or fixed cell cultures were blocked for 2 h in 5% goat serum diluted in phosphate buffered saline with 0.5% Triton (PBST, pH7.4). The samples were incubated with one primary antibody overnight at 4°C followed by incubation with secondary antibody conjugated with either Alexa488 or Alexa555 (1:3000, Invitrogen) for 2 h at room temperature. The same sections were then incubated with another primary antibody, followed by incubation with the appropriate secondary antibody. Immunohistochemistry was performed according to the procedure described previously (Liu et al., 2015). In brief, brain sections were incubated with primary antibody followed by biotinylated secondary antibodies (1:800; Jackson ImmunoResearch Laboratories). Immunosignals were visualized with 3,3-diaminobenzidine (Sigma-Aldrich).

The following primary antibodies were used: mouse anti-TH antibody (1:500, Chemicon), mouse anti-ubiqutin antibody (1:100, Santa Cruz Biotechnology), mouse anti-Parkin antibody (1:100, Santa Cruz Biotechnology), mouse anti-Flag antibody (1:100, Shanghai Genomics), rabbit anti-GFP antibody (1:1000, Invitrogen), rabbit anti-α-synuclein (1:500, Cell Signaling), mouse anti-phospho-α-synuclein (1:2000, WAKO). Sections were imaged using either a cooled CCD (DP72, Olympus) on a microscope (BX51; Olympus, Japan) or a laser confocal microscope (Leica, Bensheim, Germany). Data were obtained and processed using Adobe Photoshop 7.0 software (Adobe System).

### Cell culture and transfection

Primary cortical neurons were cultured as described previously (Martinoia et al., 2005). In brief, dissociated cortical tissue from embryonic day 18 (E18) rat pups were digested with 0.125% trypsin for 5 min at 37°C and 2 × 10^6^ neurons were transfected with 4 μg either pEGFP-C1 or pEGFP-C1-Hapln2 plasmids using a Nucleofector® II device (Amaxa). The neurons were seeded on poly-L-lysine-coated plates in Neurobasal medium (Invitrogen) containing B-27 (Invitrogen) and 2 mM Glutamax (Invitrogen).

MES23.5 cells, a gift from Dr. Weidong Le (Crawford et al., 1992), were seeded in poly-L-lysine (PLL, 10 ng/ml, Sigma)-coated plates and were incubated in DMEM/F12 medium (Invitrogen), supplemented with 5% fetal bovine serum (FBS), 2% Sato, 2 mM glutamax (Invitrogen), 1% penicillin/streptomycin (Invitrogen), and 0.1% NaHCO_3_ (Sigma-Aldrich) at 37°C in humidified air containing 5% CO_2_. HEK293T cells were maintained in DMEM containing 10% FBS at 37°C in humidified air containing 5% CO_2._ For cell transfection experiments, full-length human Hapln2 was subcloned into mammalian expression vector either pEGFP-C1 or pcDNA3.1 (Invitrogen) to produce EGFP-tagged and myc-tagged Hapln2 constructs. MES23.5 cells were transfected with either pEGFP-C1-Hapln2 or Flag-tagged Parkin, Hrd1 or Gp78 plasmids using a Nucleofector® II device (Amaxa). HEK293T cells were transiently transfected with pEGFP-C1-Hapln2 plasmid in accordance with a standard calcium phosphate protocol.

### MTT assay

The viability of MES23.5 cells was measured 24 h following the transfection of Hapln2 plasmid using the MTT [3-(4,5-dimethylthiazol-2-yl)-2,5-diphenyltetrazolium bromide] assay as described previously (Bakare et al., 2015). Briefly, MTT solution (5 mg/ml) was added to the cells in 96-well plate containing 150 μl of DMEM and incubated for 4 h at 37°C. DMSO was then added after removal of the supernatant solution and the absorbance was read on a microplate reader (Berthold Technologies) at 570 nm.

### Analysis of apoptosis using flow cytometry

The viability of MES23.5 cells was measured 24 and 48 h following the transfection of Hapln2 plasmid using flow cytometry assay. Briefly, MES23.5 cells transfected with pEGFP-C1-Hapln2 plasmid were incubated with Alex Fluor 647 (KeyGEN BioTECH)-labeled Annexin and PI (Sigma-Aldrich) for 10 min in dark and analyzed immediately by MoFlo™ XDP (Beckman Coulter).

### Statistical analysis

Statistical analysis was performed using GraphPad software (GraphPad Prism v5.0; GraphPad Software). The control group was compared with the PD group or the treatment group using student's *t*-test. *P* < 0.05 was considered as significant in statistics.

## Results

### Validation of the Hapln2 antibody

To assess the specificity of Hapln2 antibody, we transfected 293T cells with pEGFP-C1-Hapln2 plasmid (Figure S1A) and western blotting was performed using both anti-GFP antibody and anti-Hapln2 antibody. It has been reported that the predicted open reading frame of Hapln2 encoded a polypeptide of 340 amino acids containing one immunoglobulin (Ig) fold and two PTR domains with an estimated molecular weight of 38 kDa (Hirakawa et al., 2000). Western blot analysis of transfected 293T cell lysates showed that a band with the molecular weight approximately 55 kDa, corresponding to EGFP-tagged Hapln2, was detected using anti-Hapln2 antibody (Figure S1B). The same band was also recognized by anti-GFP antibody (Figure S1B), suggesting the anti-Hapln2 antibody is reliable for Western blotting. Moreover, this antibody recognized a band of approximately 48 kDa in the tissue lysates of mouse and rat SN (Figure S1C). Given that Hapln2 is one of the hyaluronan and proteoglycan binding link proteins (Spicer et al., 2003), the discrepancy in molecular weight of Hapln2 between tissue and cell lysates may result from hyaluronic acid modification.

### Hapln2 is highly expressed in DA neurons in the SN of rat

**Figure 1:**
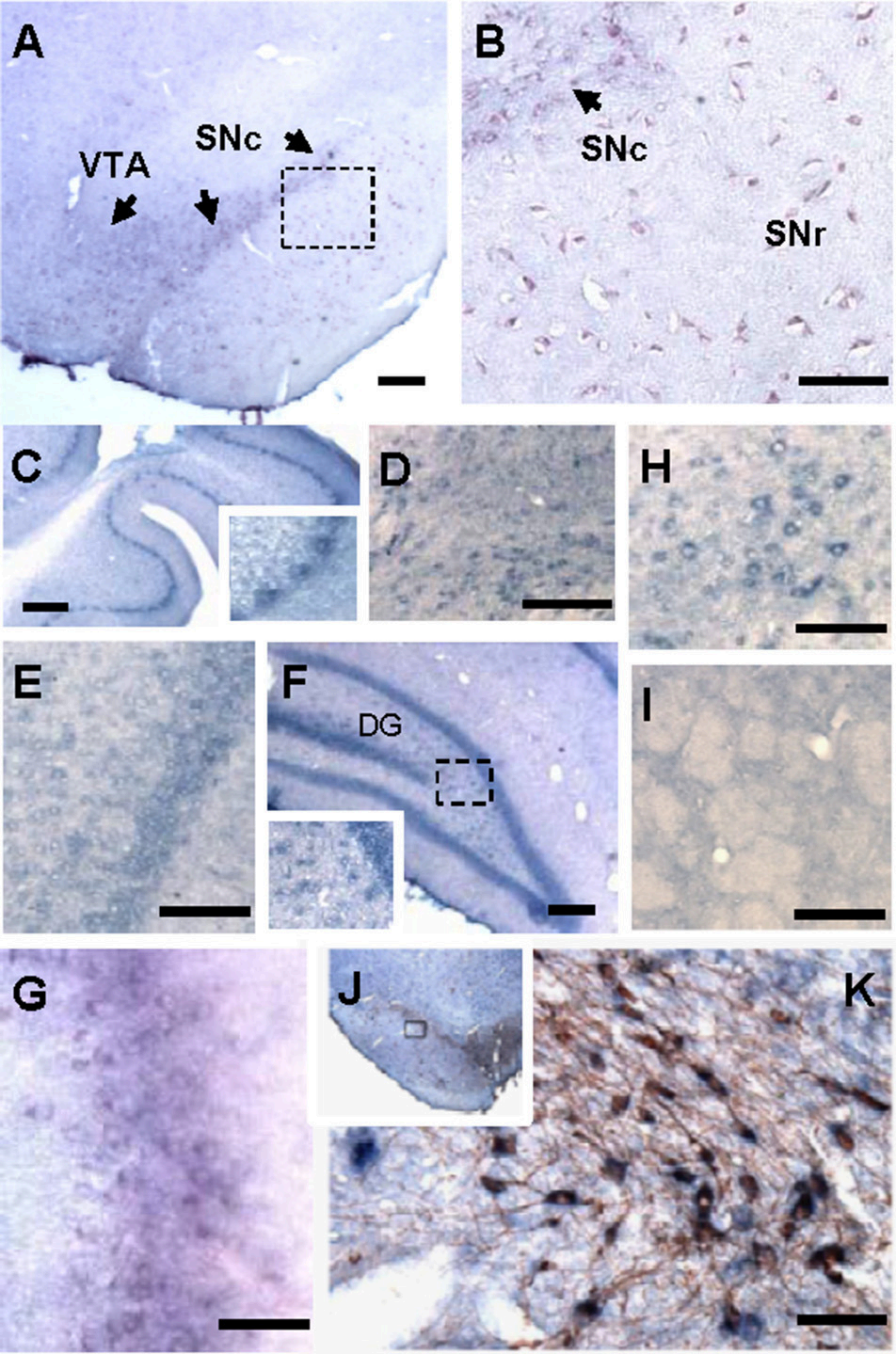
**Hapln2 is highly expressed in various brain regions including the substantia nigra of adult rat brain**. Panels **(A–H)** show that the Hapln2 hybridization signals are detected in various rat brain regions including the substantia nigra **(A,B)** and ventral tagmental area **(A)**, cerebellum **(C)**, brain stem **(D)**, cerebral cortex **(E)**, hippocampus **(F)**, olfactory bulb **(G)**, and red nucleus **(H)**. **(I)** Lower Hapln2 hybridization signals are seen in the striatum. **(J)** Representative photomicrographs showing robust Hapln2 hybridization signals in TH-positive neurons in the substantia nigra (SN). Panel **(K)** shows the enlarged images of the squared areas in J. Arrows indicate the Hapln2 hybridization signals in the VTA and SNc **(A,B)**. SNc, substantia nigra pars compacta; SNr, substantia nigra pars reticulata. Scale bars = 300 μm.

There have been only a few studies investigating the expression pattern of Hapln2 in the CNS (Hirakawa et al., 2000; Oohashi et al., 2002). Thus, the precise expression pattern of Hapln2 mRNA in adult brain is not fully established. Using ISH, we found a heterogeneous expression of Hapln2 hybridization signals in adult rat brain, with the high expression levels in both the pars compacta and pars reticulate of the SN (A9, Figures 1A,B), ventral tagmental area (VTA, A10, Figure 1A), olfactory bulb (Figure 1G), and red nucleus (Figure 1H). The former three nuclei are known as DA neuron-enriched brain regions. Robust hybridization signals were also seen in the cerebellum, brain stem, and hippocampus (Figures 1C,D,F). In contrast, relatively weak hybridization signals were observed in the cerebral cortex (Figure 1E) and no marked expression in the striatum (Figure 1I). These hybridization signals were specific for Hapln2, since there was no positive signal in negative control using a sense probe as shown in Figures S2A–D,F,G, except those in the pyramidal neurons in the hippocampus (Figure S2E).

To further determine the association between Hapln2 and nigral DA neurons, Hapln2 ISH combined with immunohistochemistry for TH was performed on adult rat brain sections. It was revealed that Hapln2 hybridization signals were highly localized to TH-positive neurons in the SN (Figures 1J,K). These results suggest that Hapln2 is widely expressed in adult brain with preferential expression in the several brain regions including the SN.

### Hapln2 expression is upregulated in the SN of PD patients

**Figure 2:**
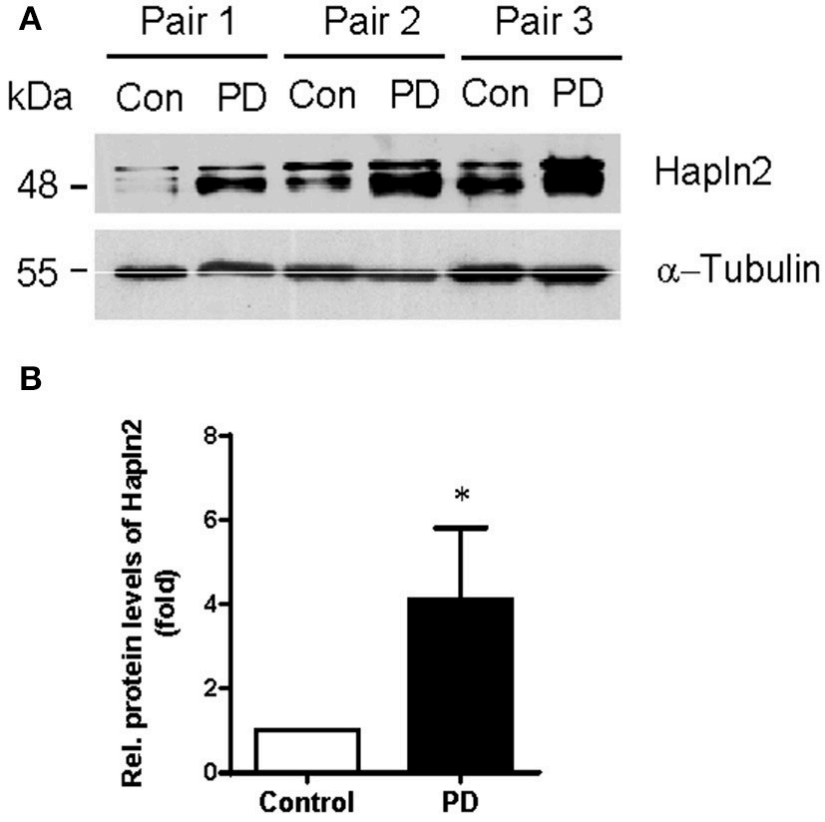
**Expression of Hapln2 protein is up-regulated in the substantia nigra of patients with PD**. **(A)** Western blotting of Hapln2 in the SN of PD patients and healthy controls. **(B)** Quantitative data shown in **(A)**. Levels of Hapln2 were normalized to actin levels. Data are expressed as mean ± S.E.M. (*n* = 3). ^*^P < 0.05 compared to control.

Our previous quantitative proteomic analysis, which was based on culture-derived isotope tags (CDIT) method and mass spectrometry, revealed that Hapln2 was one of the proteins that showed a marked increase in expression levels in the SN of the PD patients compared with the healthy controls (Liu et al., 2015). To validate whether the expression level of Hapln2 protein was truly altered in PD, we quantitatively compared the expression of Hapln2 protein in the SN in an independent cohort consisting of three pairs of PD patients and age- and gender-matched healthy controls using western blotting. Consistent with our previous findings (Liu et al., 2015), there was a profound increase in the expression of Hapln2 in the SN of patients with PD compared with healthy control subjects (Figures 2A,B).

### Hapln2 expression is upregulated in the SN of rat PD model

**Figure 3:**
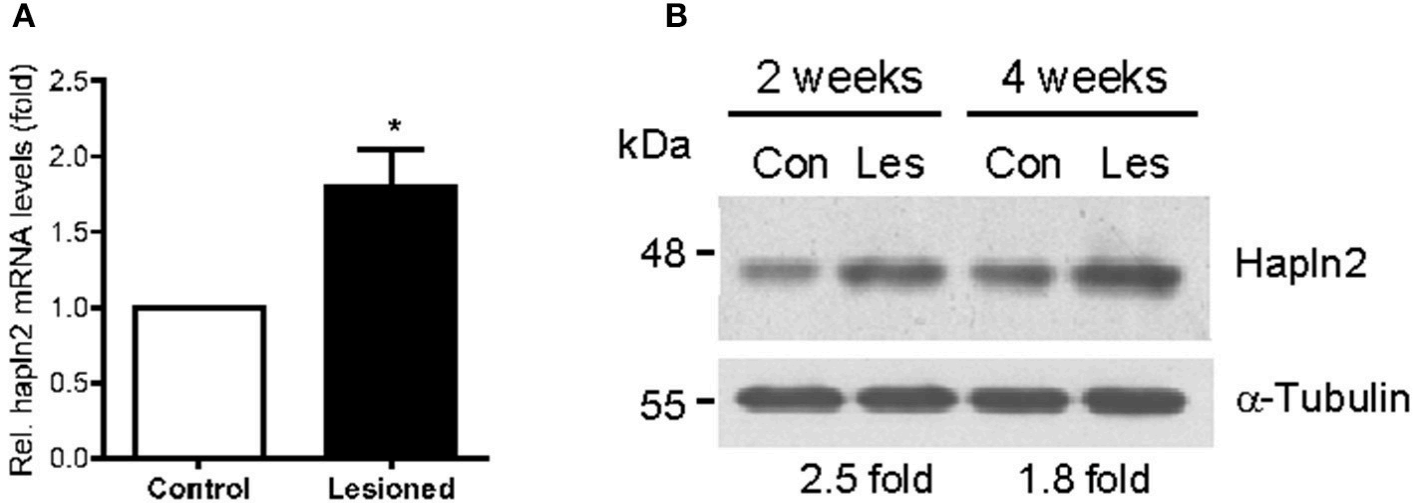
**Expression of Hapln2 is up-regulated in the substantia nigra of rat PD model. (A)** qPCR reveals the changes of Hapln2 mRNA levels in the SN of rat 2 weeks following 6-OHDA treatment. Data are expressed as mean ± S.E.M. (*n* = 3). ^*^P < 0.05. **(B)** Representative western blots showing Hapln2 expression in the substantia nigra of rat 2 and 4 weeks after 6-OHDA infusion.

Next, we determined whether the expression levels of Hapln2 protein are altered in 6-OHDA-induced rat PD model in which partial lesion of nigrostriatal DA pathway was achieved by striatal infusion. qPCR analysis showed that at 2 weeks following 6-OHDA administration, mRNA levels of Hapln2 in the lesioned SN were 1.79-fold higher than the control (Figure 3A). Moreover, there was a marked increase in expression levels of Hapln2 in the SN of 6-OHDA-treated rats compared with control 2 and 4 weeks post-lesioning (Figure 3B). These data were highly consistent with the results obtained from the SN of patients with PD (Figure 2). Taken together, these data suggest that remaining nigral DA neurons in lesioned SN may produce more Hapln2 than control during neurodegeneration.

### Overexpression of Hapln2 reduces MES23.5 cell viability

**Figure 4:**
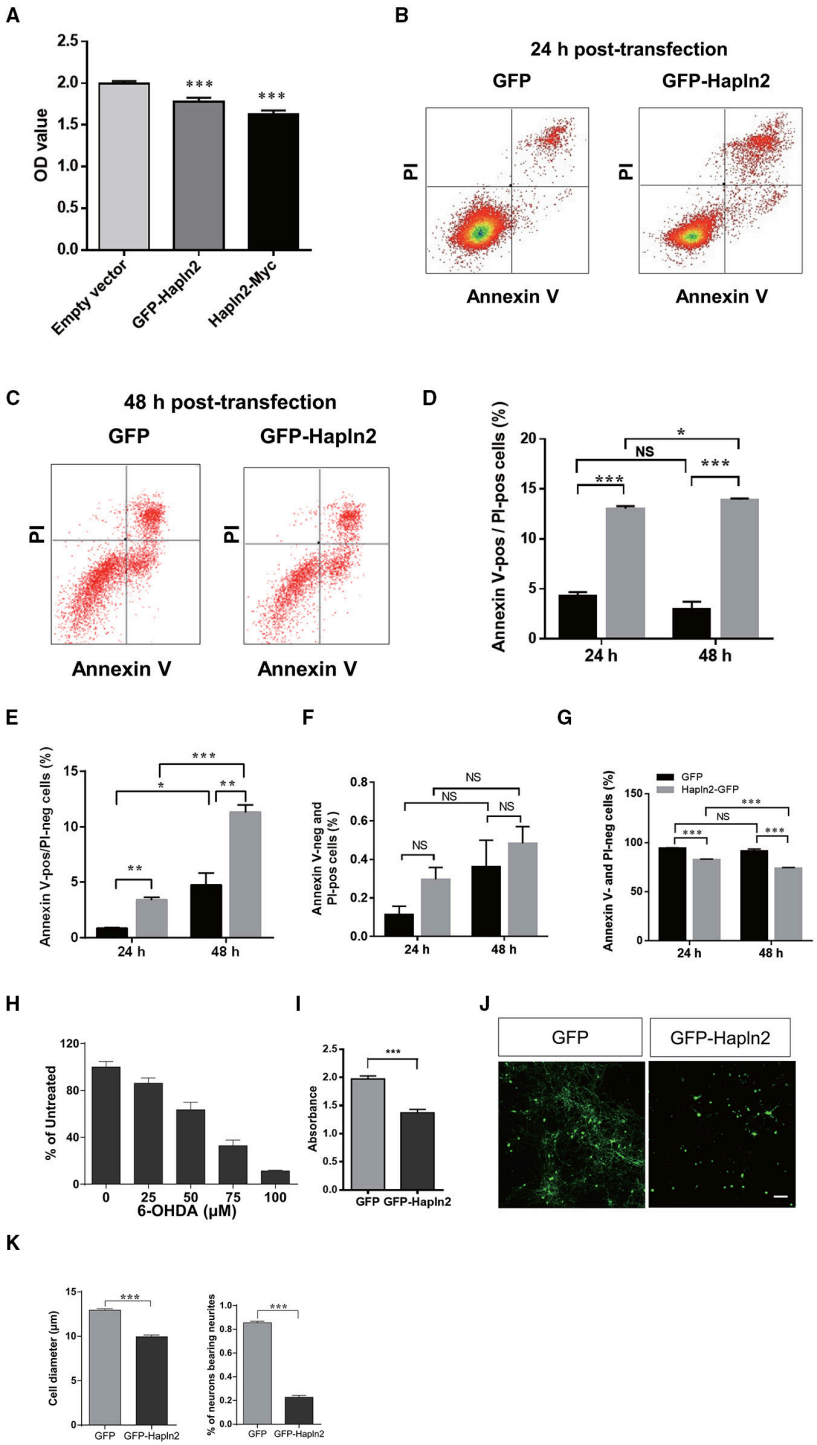
**Over-expression of Hapln2 reduces the viability of MES23.5 cell and primary cortical neurons**. **(A–C)** The viability of MES23.5 cell was evaluated by MTT assay **(A)** or flow cytometry analysis **(B,C)** 24 or 48 h after transfection with Hapln2 plasmids. **(D–G)** Quantitative data shown in **(B,C)**. **(H)** The survival ability of MES23.5 cell after 6-OHDA treatment. **(I)** The viability of MES23.5 cell transfected with Hapln2 plasmids following 6-OHDA challenge. Data are expressed as mean ± S.E.M (*n* = 6 in **A**, *n* = 3 in **D–H**, *n* = 4 in **I**). **(J)** Representative photomicrographs showing the morphology of primary cortical neurons 24 h after GFP-Hapln2 plasmids transfection. Scale bar = 20 μm. **(K)** Quantitative data of the neuronal soma diameter and the percentage of neurons bearing neurites in **(J)**. Data are expressed as mean ± S.E.M (*n* = 3). ^*^p < 0.05, ^**^p < 0.01, ^***^p < 0.001.

To evaluate the impact of Hapln2 overexpression to DA cells, the MES23.5 cell viability was assessed using MTT assay following transfection of the plasmids encoding either GFP-Hapln2 or myc-tagged Hapln2 plasmids. It was found that the viability of MES23.5 cell was markedly reduced in MES23.5 cells overexpressing either GFP- or myc-tagged Hapln2 compared with control group (Figure 4A). Moreover, flow cytometry analysis showed that overexpression of Hapln2 resulted in approximately four-fold increases in the percentage of both Annexin V- and PI-positive cells or Annexin V-positive but PI-negative cells 24 h after Hapln2 plasmid transfection, suggesting that Hapln2 overexpression results in apoptosis or necrosis (Figures 4B–E). Interestingly, at 48 h after the transfection, apoptosis (Annexin V-positive/PI-negative cells) of MES23.5 cells transfected with Hapln2-GFP was more severe (GFP: 3.42 ± 0.191% vs. Hapln2-GFP: 11.36 ± 0.63%, Figures 4B–E). While there was no significant alteration in the percentage of Annexin V-negative but PI-positive cells 24 or 48 h after Hapln2-GFP transfection compared with control (Figure 4F). Accordingly, the percentage of viable cells (both Annexin V- and PI-negative cells) was decreased from 94.66 ± 0.35% to 83.24 ± 0.31% 24 h after transfection and from 91.91 ± 1.84% to 74.24 ± 0.68% 48 h after transfection, while there was no marked changes in control over time (Figure 4G).

Moreover, we determined the influence of Hapln2 overexpression on MES23.5 cell viability in the presence of neurotoxin 6-OHDA. Treatment of MES23.5 cells with 6-OHDA dramatically reduced their survival in a concentration-dependent manner. We selected concentration of 50 μM 6-OHDA for further experiments. At this concentration of 6-OHDA, there was 37% reduction of cell viability (Figure 4H). Exposure of MES23.5 cells overexpressing Hapln2 to 50 μM 6-OHDA for 24 h resulted in 31% reduction in cell viability compared to those transfected with an empty vector (Figure 4I). To further evaluate the potential impact of Hapln2 on neuronal cell survival, we transfected primary cortical neurons with an expression construct for Hapln2-GFP. Overexpression of Hapln2-GFP resulted in marked decreases in the diameter of tectal neuronal soma (GFP: 12.95 ± 0.1483 vs. Hapln2-GFP: 9.962 ± 0.1854 μm) and the percentage of neurons bearing neurites (GFP: 85.56 ± 1.20% vs. Hapln2-GFP: 22.75 ± 1.60%) 24 h after transfection (Figures 4J,K). These data suggest that overexpression of Hapln2 is detrimental to DA neurons.

### Overexpression of Hapln2 promotes protein aggregation

**Figure 5:**
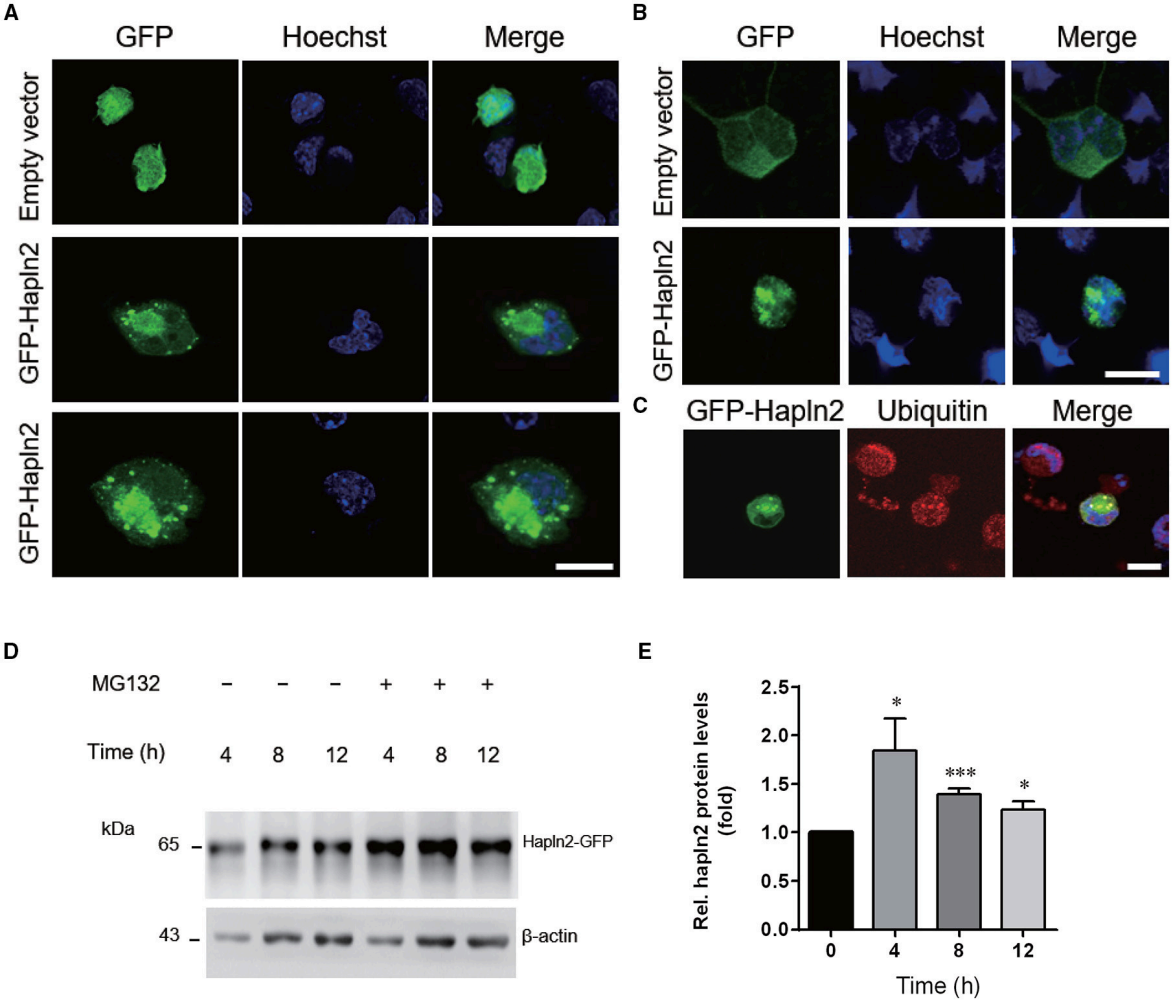
**Hapln2 is associated with ubiquitin-proteasome pathway (UPP)**. **(A)** Confocal images of Hapln2 aggregates in MES23.5 cells transfected with Hapln2-GFP plasmids. Scale bar = 10 μm. **(B)** Representative photomicrographs showing Hapln2 aggregates in primary cortical neurons transfected with GFP- Hapln2 plasmids. Scale bar = 10 μm. **(C)** Immunofluorescent staining for ubituitin in MES23.5 cells transfected with Hapln2-GFP plasmids. Scale bar = 10 μm. **(D)** Expression levels of Hapln2-GFP was analyzed in MES23.5 cells transfected with the Hapln2-GFP plasmid at indicated times after MG132 (20 μM) treatment. **(E)** Quantitative data of Hapln2 expression in **(D)** (*n* = 4). ^*^p < 0.05, ^***^p < 0.001.

To further understand the potential function of Hapln2 on DA degeneration, we examined the subcellular structures in individual MES23.5 cells and primary cortical neurons overexpressing GFP-Hapln2. Under basal conditions, the pEGFP-transfected cells presented no GFP aggregates 24 h post-transfection. However, significant elevations in protein aggregates were detected in MES23.5 and primary neurons transfected with GFP-Hapln2 plasmids (Figures 5A,B). To elucidate the characteristics of aggregates formed, we conducted immunostaining using antibodies against ubiquitin. It has been widely recognized that ubiquitin-proteasome system (UPP) is one of the main pathways for degradation of aggregated or misfolded proteins in eukaryotic cells (Koshizuka et al., 2015; Long et al., 2015). Ubiquitin is one of the components of Lewy bodies, indicating the important role of UPP in PD (Cai et al., 2015; Navarro-Yepes et al., 2015). It was shown that aggregated Hapln2 protein was co-localized with ubiquitin (Figure 5C), suggesting that the aggregated Hapln2 was ubiquitinated. Consistent with these results, MG132 treatment also induced profound upregulation of the exogenous GFP-tagged Hapln2 (Figures 5D,E), suggesting that the degradation of Hapln2 is dependent on UPP.

### E3 ubiquitin ligases are co-localized with Hapln2 in cytoplasmic aggregates

**Figure 6:**
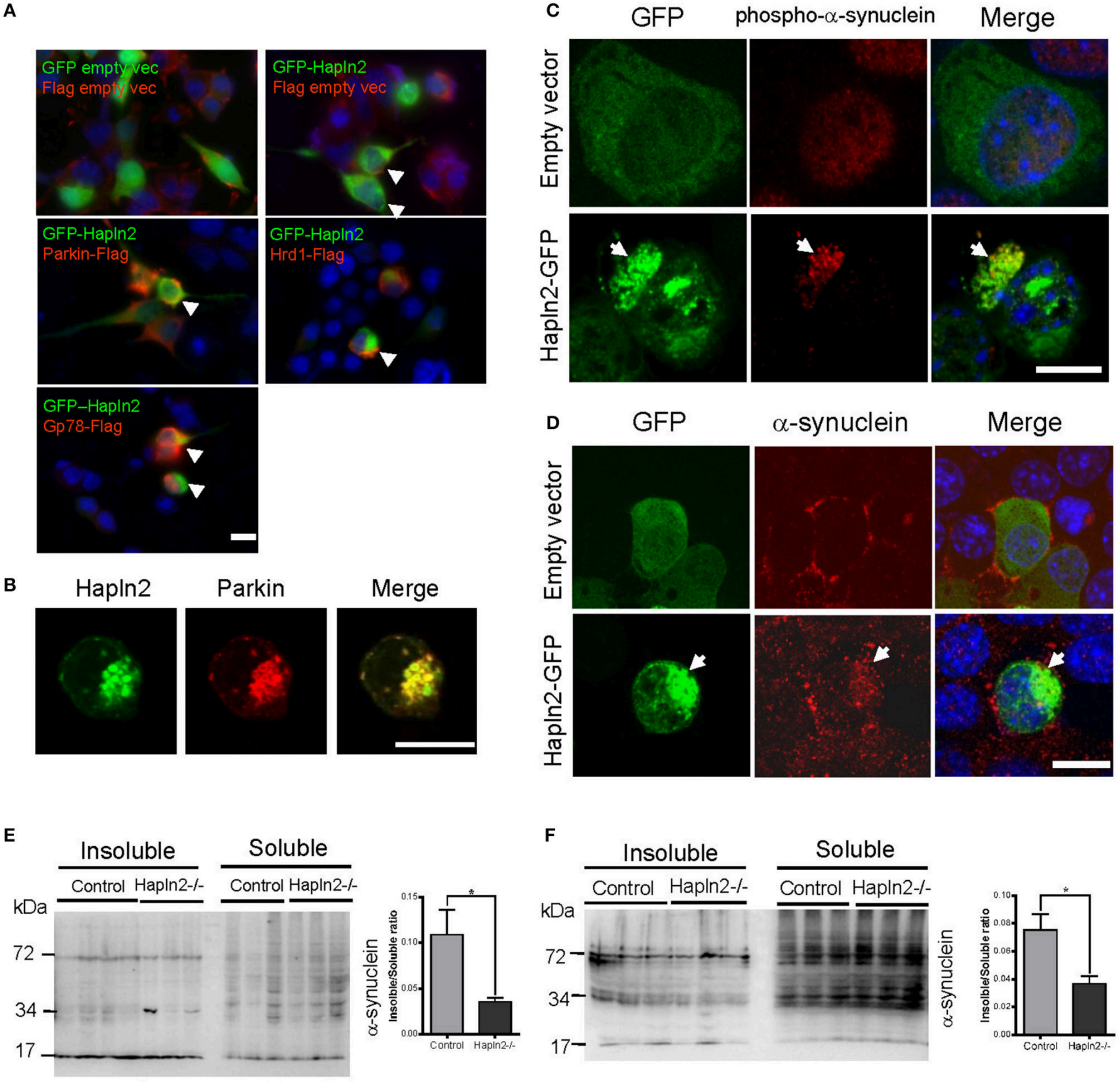
**Exogenous Hapln2-GFP is co-localized with E3 ubiquitin ligases and α-synuclein**. **(A)** Immunofluorescent staining co-localization of Hapln2 and E3 ubiquitin ligases including Parkin, Hrd1, Gp78 *in vitro.* Arrow heads point to the merged cells. **(B)** Immunofluorescent staining showing the co-localization of Hapln2 with Parkin (C418R). **(C,D)** Representative micrographs showing co-localization of Hapln2-GFP and phosph-α-synuclein **(C)** or α-synuclein **(D)** in MES23.5 cells transfected with Hapln2-GFP plasmids. Arrows indicate the colocalization of Hapln2 and phospho-α-synuclein **(C)** or α-synuclein **(D)**. Scale bars = 10 μm. **(E,F)** Expression levels of RIPA-soluble and urea-soluble protein fractions extracted from SN **(E)** and cerebellum **(F)**. Data are expressed as mean ± S.E.M. (*n* = 3). ^*^p < 0.05.

E3 ubiquitin ligases (E3s) are known to play a key role in determining substrate specificity and catalyzing the transfer of ubiquitin from E2 enzymes to the substrate in the UPP (Soucy et al., 2009; Metzger et al., 2012; Uchida and Kitagawa, 2015). To determine whether E3 ubiquitin ligases are involved in the ubiquitination of Hapln2-containing protein aggregates, we investigated the expression pattern of ubiquitin ligases Parkin, Hrd1, and Gp78 in MES23.5 cells overexpressing GFP-Hapln2. It was revealed that approximately 20% of the cells co-transfected with GFP-Hapln2 and Parkin showed co-localization of Hapln2 with Parkin in intracellular aggregates (Figure 6A). Likewise, similar percentage of co-transfected cells showed co-localization of Hapln2 with either Hrd1 or Gp78 in the protein aggregates, while there was only 3% of cells co-transfected with the plasmids encoding GFP-Hapln2 and FLAG-empty vector showed Hapln2-positive aggregates and none aggregates was seen in cells co-transfected with both GFP and FLAG empty vectors which served as negative controls (Figure 6A). These data suggest that Hapln2 may promote sequestration of E3 ligases by recruiting them into the aggregates.

### Aggregated Hapln2 sequestrates parkin (C418R) and α-synuclein

We then asked a question whether Hapln2 has relevance with human mutant genes causing PD. It has been demonstrated that the RING domains of Parkin is essential for its E3 ubiquitin ligase activity and the mutation (C418R) in the RING domains of Parkin resulted in the aggregation of Parkin (Gu et al., 2003). Thus, we examined whether the Parkin mutant is involved in the ubiquitination of Hapln2. Immunofluorescent staining showed that Hapln2 was also co-localized with the C418R missense mutant form of Parkin in insoluble aggregates in some of the transfected cells (Figure 6B), indicating that Hapln2 is associated with protein aggregation caused by Parkin mutation.

α-Synuclein is a major component of Lewy bodies, a key pathological characteristic of PD (Zhang et al., 2005). We examined whether the overexpressed Hapln2 is associated with α-synuclein. It was found that exogenous Hapln2 was co-localized with endogenous phospho-α-synuclein and α-synuclein in the aggregates in MES23.5 cells (Figures 6C,D), suggesting that Hapln2 overexpression perturbed the intracellular distribution of α-synuclein. To further clarify the role of Hapln2 in protein aggregation, we isolated the RIPA-soluble and RIPA-insoluble protein fractions from the SN and cerebellum of Hapln2 knockout mice. Western blotting analysis showed that significant decreases in the ratio of insoluble to soluble α-synuclein was observed in both the SN and cerebellum of Hapln2-deficient mice compared with control (Figures 6E,F), suggesting that absence of Hapln2 compromise α-synuclein-associated protein aggregation. Taken together, these results indicate that the upregulated Hapln2 protein may contribute to PD pathology by enhancing α-synuclein accumulation in DA neurons.

## Discussion

In the present study, we described Hapln2 expression pattern in adult rat brain and, interestingly, the upregulation of Hapln2 expression levels in the SN of patients with PD as well as rat PD model. Hapln2 overexpression resulted in the formation of cytoplasmic aggregates in primary cortical neurons and MES23.5 cell. Moreover, Hapln2 overexpression led to a marked reduction of DA cell viability and exacerbated 6-OHDA-induced loss of MES23.5 cell viability. Hapln2 overexpression also promoted α-synuclein accumulation in MES23.5 cell and ablation of Hapln2 resulted in a decrease in the levels of α-synuclein in insoluble fraction in brain tissues. These findings highlight the importance of Hapln2 as a novel factor contributing to neurodegeneration in PD.

### Expression of Hapln2 in nigral DA neurons

Previous studies have demonstrated that the Hapln2 immunosignals were primarily localized in myelinated fiber tracts in the adult brain and were co-expressed with the versican V2 isoform at the nodes of Ranvier (Oohashi et al., 2002). These data imply that Hapln2 may play an important role in the formation of an ion diffusion barrier at the nodes promoting neuronal conduction. In the present study, we found that Hapln2 mRNA is expressed in a variety of brain regions, with moderate expression in the cerebral cortex, where is enriched with myelinated axons from pyramidal neurons. This supports the idea that Hapln2 may play an important role in the formation of an ion diffusion barrier at the nodes in the myelinated neurons as previously indicated. It is interesting to note, however, that Hapln2 mRNA is also expressed in some of other brain regions, where the neurons usually have no myelination, for example, the dendate gyrus in the hippocampus and substantia nigra (Figure 1). These data indicate that Hapln2 may play distinct roles in these brain regions from what was previously reported.

### Hapln2 as a new contributor to PD pathology

We found in the present study that protein levels of Hapln2 were robustly upregulated in nigral tissue of PD patients and PD animal models. To investigate the consequence of upregulated expression of Hapln2, Hapln2 was overexpressed in MES23.5 cells, a dopaminergic cell line. Exogenous Hapln2 appeared as cytoplasmic aggregates in MES23.5 cells, and the aggregates were co-localized with ubiquitin, indicating the possible relationship between Hapln2 and ubiquitin degradation pathway. Degradation of a target protein by UPP requires a series of reactions which involve three key enzymes including Ub-activating enzyme (E1), Ub-conjugating enzyme (E2), and Ub-ligase (E3) (Weissman, 2001; Papaevgeniou and Chondrogianni, 2014). Among these ubiquitin related enzymes, E3s play a crucial role in catalyzing the ubiquitin chains on the specific substrate (Long et al., 2015). Previous research has reported that ubiquitination of misfolded proteins is also a common hallmark of a variety of age-related neurodegenerative diseases including PD (Zhang et al., 2005; Bhat et al., 2014; Hromadkova et al., 2015; Squitieri and de Yebenes, 2015). And it has been reported that dysfunction of UPP induced by proteasome inhibitors, such as MG132 and epoxomicin, led to dopaminergic degeneration in both cell cultures and animal models (Kikuchi et al., 2003; Sun et al., 2006), indicating the importance of UPP in PD. In the present study, MG132 treatment induced the upregulation of exogenous Hapln2 in MES23.5 cells, indicating that Hapln2 may be degraded by ubiquitin degradation pathway.

Our previous proteomics analysis revealed that the upregulation of Hapln2 showed the highest fold change in the brain of PD patients (Liu et al., 2015). These observations along with the data shown in Figure 2 in the present study strongly suggest a close correlation between Hapln2 and PD. However, the biological role and significance of this change remain to be clarified. Our data suggest that upregulation of Hapln2 is associated with abnormal protein aggregation, based on the observations that exogenous Hapln2 was co-localized with the E3 ligases, including Parkin, Hrd1, and Gp78. Parkin is a PD-associated gene which encodes an E3-ubiquitin ligase (Kitada et al., 1998). The finding that exogenous Hapln2 was colocalized with Parkin C418 suggests that dysfunction of Parkin is associated with Hapln2 degradation. Another ubiquitin ligase Hrd1 has been reported to promote the ubiquitination and degradation of Parkin-associated endothelin receptor-like receptor (Pael-R), which is an ER stress inducer contributing to familial PD (Omura et al., 2013). Besides, Hrd1 could suppress the neuronal cell death caused by 6-OHDA (Omura et al., 2013), suggesting the potential role of Hrd1 in PD pathogenesis. Earlier work also showed another E3 ubiquitin ligase Gp78 could promote the degradation of misfolded protein regulated by Hrd1 (Zhang et al., 2015).

Given that LB is one of the hallmarks of PD pathology (Zhang et al., 2005) and strong correlation between Hapln2 and the “molecular signature” of LB such as α-synuclein and E3s shown in the present study, we speculate that Hapln2 could be one of components of LB. There is evidence that LB is consisted of a heterogeneous mixture including lipids forming the core of the inclusions and proteins mainly including neurofilament, various proteasomal elements, and α-synuclein (Wakabayashi et al., 2013; Araki et al., 2015; Power et al., 2015). Our result in the present study showed that α-synuclein could be sequestered by Hapln2 aggregates and Hapln2 deficiency altered the levels of α-synuclein in insoluble fraction (Figure 6). Although there is no direct pathological evidence that Hapln2 is a integral constituent of LB in PD, Hapln2 has been recently shown to be accumulated in the neurofibrillary tangle of Alzheimer's brain (Bandopadhyay, 2016), indicating that Hapln2 can be the part of protein deposition process involved in pathogenesis of both AD and PD. Future study is needed to determine the role of Hapln2 in the formation of LB.

In conclusion, as a novel brain specific-hyaluronan and protoglycan link protein, the knowledge of the function of Hapln2 in neurological diseases is still lacking. In the present study, we have shown that Hapln2 was involved in the pathogenesis of PD, which will not only promote our understanding of the role of Hapln2 in the PD pathogenesis but also provide new insights into pathogenesis of age-related neurodegenerative disorders.

## Author contributions

QW, QZ conducted most of the *in vivo* and *in vitro* experiments and the data analysis; SZ, WS contributed to pilot experiments, YY, YL, and JH conducted genotyping; YY, XZ, YG, contributed to cell cultures; SZ, XG, XW contributed to the discussion; JZ supervised the project and wrote the manuscript.

### Conflict of interest statement

The authors declare that the research was conducted in the absence of any commercial or financial relationships that could be construed as a potential conflict of interest.

## Supplementary material

Figure S1 **Verification of the specificity of Hapln2 antibody. (A)** Representative photomicrographs showing the expression pattern of Hapln2-GFP in HEK293T cells transfected with Hapln2-GFP plasmids. **(B)** Western blot analysis of cell lysates of 293T cells transfected with Hapln2-GFP plasmids using anti-GFP and anti-Hapln2 antibodies. **(C)** Western blot analysis of tissue lysates of the substantia nigra of adult rat using anti-Hapln2 antibody.

Figure S2 **Verification of the specificity of Hapln2 *in situ* hybridization probe**. Panels **(A–G)** show the hybridization signals of Hapln2 in ventral midbrain **(A)**, cerebral cortex **(B)**, striatum **(C)**, cerebellum **(D)**, hippocampus **(E)**, olfactory bulb **(F)**, and brain stem **(G)** of rat slices using a sense probe. Scale bar = 300 μm.
