# Supplementary figures and images for: Elevated Hapln2 Expression Contributes to Protein Aggregation and Neurodegeneration in an Animal Model of Parkinson's Disease
Source: Front Aging Neurosci. 2016 Aug 23;8:197. doi: 10.3389/fnagi.2016.00197 (PMC4993759; doi:10.3389/fnagi.2016.00197)

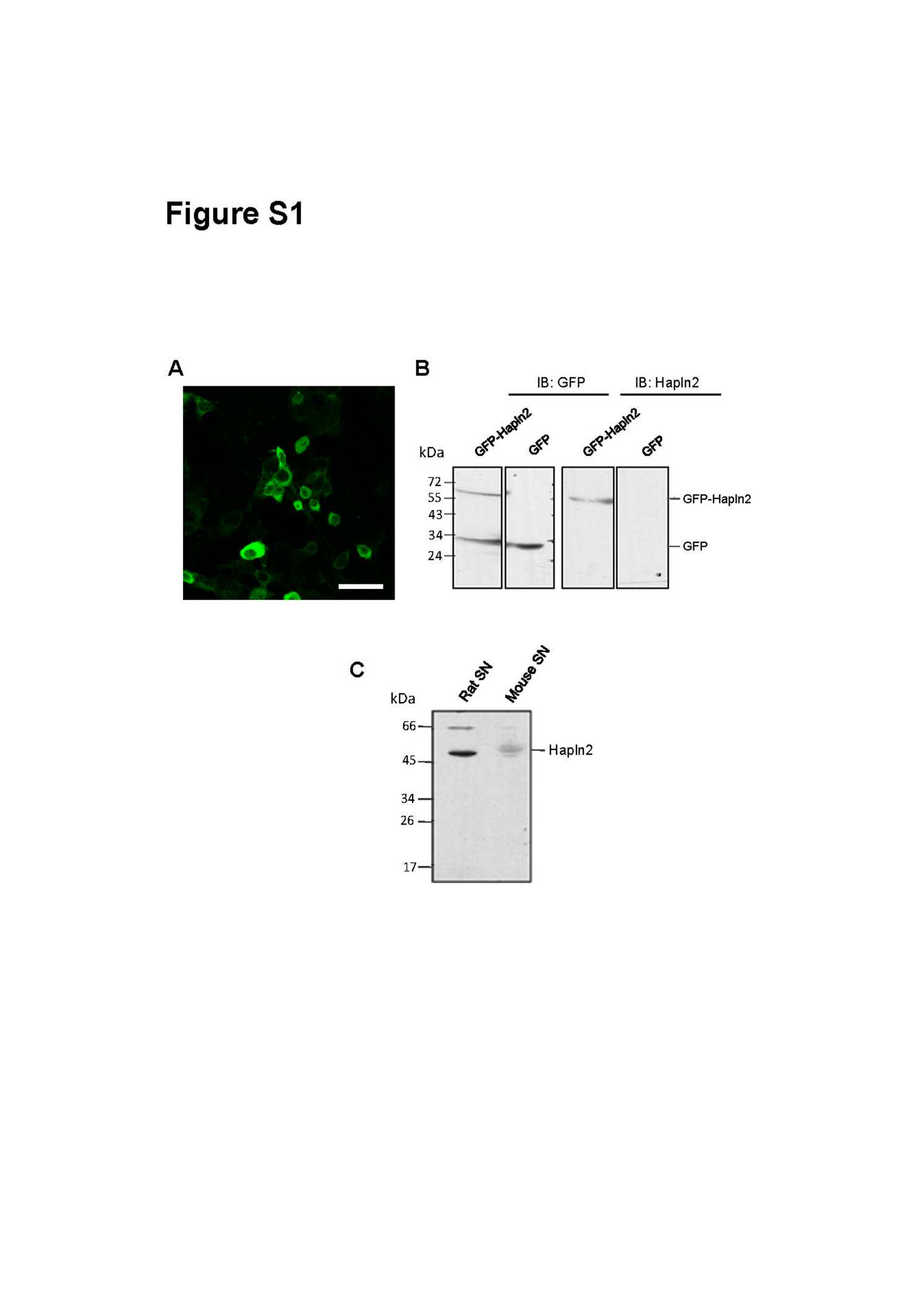

Supplement: Figure S1 — Verification of the specificity of Hapln2 antibody. (A) Representative photomicrographs showing the expression pattern of Hapln2-GFP in HEK293T cells transfected with Hapln2-GFP plasmids. (B) Western blot analysis of cell lysates of 293T cells transfected with Hapln2-GFP plasmids using anti-GFP and anti-Hapln2 antibodies. (C) Western blot analysis of tissue lysates of the substantia nigra of adult rat using anti-Hapln2 antibody. [file Image1.TIFF]

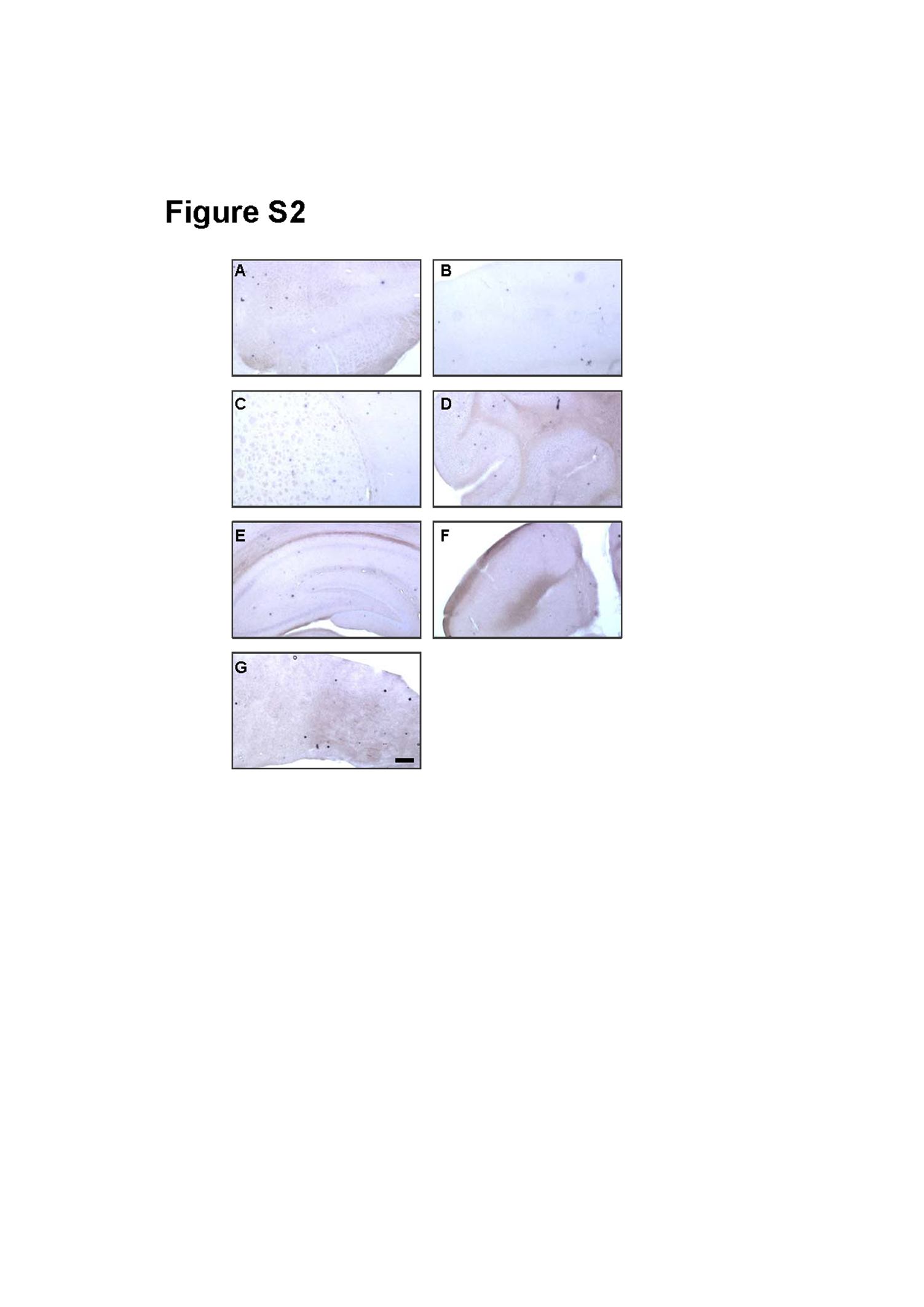

Supplement: Figure S2 — Verification of the specificity of Hapln2 in situ hybridization probe. Panels (A–G) show the hybridization signals of Hapln2 in ventral midbrain (A), cerebral cortex (B), striatum (C), cerebellum (D), hippocampus (E), olfactory bulb (F), and brain stem (G) of rat slices using a sense probe. Scale bar = 300 μm. [file Image2.TIFF]
